# Determination of the energy band gap of Bi_2_Se_3_

**DOI:** 10.1038/s41598-017-07211-x

**Published:** 2017-07-31

**Authors:** G. Martinez, B. A. Piot, M. Hakl, M. Potemski, Y. S. Hor, A. Materna, S. G. Strzelecka, A. Hruban, O. Caha, J. Novák, A. Dubroka, Č. Drašar, M. Orlita

**Affiliations:** 1 Laboratoire National des Champs Magnétiques Intenses, CNRS-UGA-UPS-INSA-EMFL, 25, avenue des Martyrs, 38042 Grenoble, France; 2 Department of Physics, Missouri University of Science and Technology, Rolla, MO 65409 USA; 3 Institute of Electronic Materials Technology, ul. Wolczynska 133, 01-919 Warsaw, Poland; 4 Institute of Physics, Polish Academy of Science, Warsaw, PL-02668 Poland; 5 CEITEC MU, Masaryk University, Faculty of Science, 61137 Brno, Czech Republic; 6 Institute of Applied Physics and Mathematics, Faculty of Chemical Technology, University of Pardubice, Studentská 84, CZ-532 10 Pardubice, Czech Republic; 7 Institute of Physics, Charles University in Prague, CZ-121 16 Prague, Czech Republic

## Abstract

Despite intensive investigations of Bi_2_Se_3_ in past few years, the size and nature of the bulk energy band gap of this well-known 3D topological insulator still remain unclear. Here we report on a combined magneto-transport, photoluminescence and infrared transmission study of Bi_2_Se_3_, which unambiguously shows that the energy band gap of this material is direct and reaches *E*_*g*_ = (220 ± 5) meV at low temperatures.

## Introduction

The existence of the energy band gap, separating the conduction and valence bands, is a key characteristic of all topological insulators, which allows these materials to behave as insulators in interior, but still, to conduct electric current via specific, topologically protected states on their surfaces^1, 2^.

In bismuth selenide (Bi_2_Se_3_) – perhaps the most representative example of 3D topological insulators – the band gap is located at the Brillouin zone center, however, the consensus regarding further details, notably its size and nature, has not yet been established. Rather large values for the band gap (above 300 meV) have been deduced using surface-sensitive techniques, ARPES and STM/STS^3–5^, which also often (but not always)^6, 7^ imply its indirect nature related to the pronounced “camelback” profile of the valence band. In contrast, optical experiments^8–11^ consistently show a direct band gap around 200 meV. Importantly, the missing consensus about the band gap is not a minor drawback in our understanding properties of Bi_2_Se_3_. It is the size and nature of the band gap which are the parameters needed for interpretation of ARPES data^3, 4^, most notably, for the correct positioning of the surface cone with respect to the bulk bands. For a wider band gap, the Dirac point of the surface states approaches the midgap position. A narrower band gap shifts the charge neutrality point towards the valence band, which may, for instance, explain the pronounced electron-hole asymmetry of the surface cone observed in STM/STS experiments^12^.

In this paper, we address the existing controversy about the band gap of Bi_2_Se_3_ using an experimental approach which combines optical methods – infrared transmission and photoluminescence – with magneto-transport, successfully used in the past also for other materials, see, e.g., refs 13 and 14. We unambiguously show that the energy band gap of Bi_2_Se_3_ is direct and reaches *E*_*g*_ = (220 ± 5) meV.

## Experimental Details

To determine the band gap of Bi_2_Se_3_, bulk crystal of this compound was grown using the modified Bridgman method where stoichiometric mixture of high purity Bi and Se elements were vacuum sealed in a quartz tube, heated up to the melting point and cooled down to room temperature with the rate of 0.1 °C/min under the temperature gradient of about 10 °C/cm along the tube length in a box furnace. As-grown crystals showed a strong *n*-type doping (close to 10^19^ cm^−3^) due to selenium vacancies, which was reduced by the after-growth annealing in selenium vapors down to 10^18^ cm^−3^, nevertheless with a certain variation of the electron density across the crystal.

**Figure 1:**
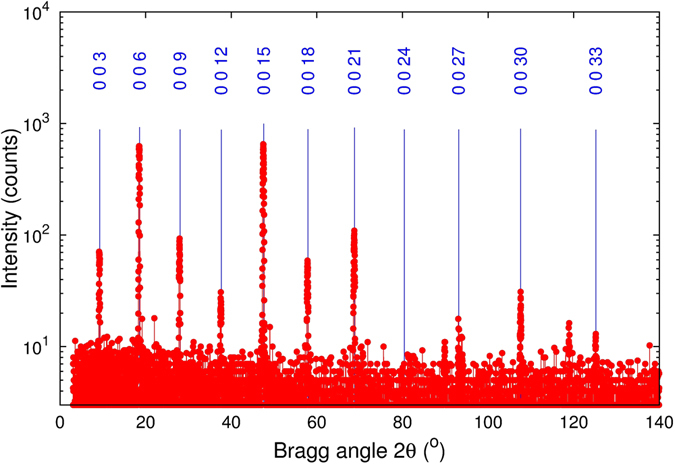
XRD symmetric scan collected from a Bi_2_Se_3_ sample. Blue lines denote theoretical positions of the diffraction peaks according to the structural database^15^.

The bulk crystal was characterized using x-ray diffractometer equipped with Cu x-ray tube, channel-cut germanium monochromator and scintillation detector. The standard *θ *− 2*θ* scan is shown in Fig. 1. The observed x-ray diffraction peaks correspond well to the c-lattice parameter of *c* = (28.64 ± 0.01) Å in an perfect agreement with tabulated value 28.636 Å^15^. The crystal has been then sliced using microtome machine perpendicular to the *c*-axis of Bi_2_Se_3_. Two free-standing layers with the thicknesses of *d* = 6.5 and 10 *μ*m have been chosen for this study denoted as samples A and B, respectively. They were explored in low temperature photoluminescence (PL), infrared transmission and magneto-transport experiments.

To measure PL spectra, the samples were placed in a helium bath cryostat and excited by *λ* = 660 nm diode laser with an approximate power of 100 *μ*W focused on spot of 1 mm^2^. The collected signal was delivered to a Fourier transform spectrometer, analyzed and detected by a liquid-nitrogen-cooled MCT detector. To measure infrared transmission, a macroscopic area of the sample (≈3 mm^2^) was exposed to the radiation of a globar, which was analyzed by a Fourier transform spectrometer, using light-pipe optics delivered to the sample placed in a helium bath cryostat and detected by a composite bolometer placed just below the sample. Magneto-transport experiments were conducted on samples contacted using silver paste in the Van der Pauw-like geometry. Measurements were performed using a standard low-frequency lock-in technique in a variable temperature insert, with the magnetic field applied along the *c*-axis of Bi_2_Se_3_.

## Discussion

**Figure 2:**
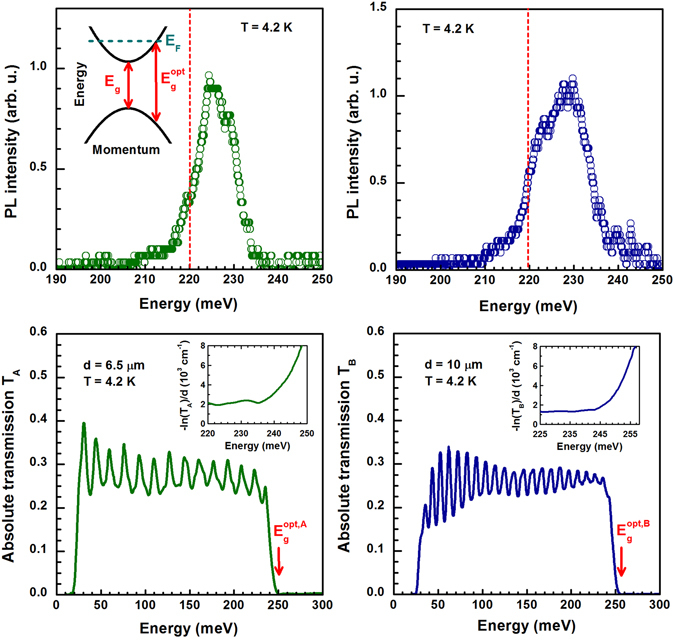
(**a**,**b**) Low-temperature PL spectra collected from samples A and B, respectively. The dashed vertical lines denote the estimated energy of the band gap, see the discussion in the main text. The schematic band structure of Bi_2_Se_3_ is plotted in the inset of the part (**a**), the difference between *E*_*g*_ and ${E}_{g}^{{\rm{opt}}}$ corresponds to the Moss-Burstein shift. (**c**,**d**): Infrared transmission data *T*_*A*_ and *T*_*B*_ taken on samples A and B, respectively. The corresponding absorbance spectra, −ln(*T*)/*d*, around the interband absorption edge ${E}_{g}^{{\rm{opt}}}$, normalized by the sample thickness, are plotted in the insets. The pronounced modulation of the transmission spectra is due to Fabry-Pérot oscillations, which show rather high crystalline quality of the studied Bi_2_Se_3_ bulk samples and which allows us to estimate, knowing the thickness of the samples, the refraction index: *n* ≈ 5.5–6.

We start the discussion with the PL spectra recorded from both samples at liquid-helium temperatures (Fig. 2a and b). It is just the existence of a well-defined efficient PL emission, which clearly indicates a direct nature of the band gap in Bi_2_Se_3_. In other words, the conduction-band electrons are located around the same point of the Brillouin zone as the photo-excited holes in the valence band. The positions of the observed PL emission in spectra then provide us with the very first estimate for the size of the band gap.

In the simplest possible scenario, one may assume that the the observed PL corresponds to a direct band-to-band (vertical in *k*-space) recombination of electrons from the degenerate gas at the bottom of the conduction band with photo-excited holes at the top of the valence band. In such a case, the band gap can be associated with the inflection point at the low-energy onset of PL emission line, having for both samples the energy close to 220 meV and denoted in Fig. 2a and b by vertical dashed lines. From other, more complex scenarios for PL mechanisms, we may exclude, due to screening effects in the degenerate electron gas, the excitonic-like recombination. However, one may still imagine a variety of defects-related radiative recombination channels, often efficient in semiconducting compounds and giving rise to sub-band-gap emission of light. The photon energy of the PL emission thus serves only as a lower bound for the (direct) band gap in Bi_2_Se_3_.

Another estimate of the band gap *E*_*g*_, in this case implying its upper bound, comes from the analysis of the infrared transmission (Fig. 2c and d). Both samples show rather broad transparency window, which is at low photon energies limited by the free carrier response (reflectivity below the plasma edge) and also by the absorption due to infrared active phonons^16^. At high photon energies, the transmission window closes due to a relatively sharp onset of interband absorption. This onset is often referred to as the optical band gap ${E}_{g}^{{\rm{opt}}}$ and represents the upper bound for the band gap due to the well-known Moss-Burstein shift that is characteristic of semiconductors with a degenerate electron or hole gas (see the inset of Fig. 2a)^17^.

The approximate position of the optical band gap is denoted in Fig. 2c and d by the vertical arrow. A more precise read-out of ${E}_{g}^{{\rm{opt}}}$ is possible when transmission is plotted as absorbance and normalized by the sample thickness (insets of Fig. 2c,d). When the optical band gap is approached, the absorbance becomes dominantly governed by absorption: α ≈−ln(*T*)/*d*, which increases almost exponentially that is reminiscent of the Urbach edge absorption in (undoped) semiconductors^18^. The optical band gap may then be associated with the photon energy at which the absorption coefficient *α* approaches 10^4^ cm^−1^, a value typical for interband absorption in direct-band-gap semiconductors^19^. For the sample A and B, we obtain ${E}_{g}^{\text{opt},{\rm{A}}}=\mathrm{(250}\pm \mathrm{3)}$ meV and ${E}_{g}^{\text{opt},{\rm{B}}}=\mathrm{(258}\pm \mathrm{3)}$ meV, respectively.

To extract the size of the band gap *E*_*g*_ from ${E}_{g}^{{\rm{opt}}}$, the Moss-Burstein shift has to be estimated. In a degenerate *n*-type semiconductor with a direct band gap, this shift reads: ${\rm{\Delta }}{E}_{{\rm{MB}}}={E}_{g}^{{\rm{opt}}}-{E}_{g}=\mathrm{(1}+{m}_{e}/{m}_{h}){E}_{F}$, where *E*_*F*_ is the Fermi energy and *m*_*e*(*h*)_ stands for the electron (hole) effective mass. The anisotropy of effective masses enters this expression only, when the ratio *m*_*e*_/*m*_*h*_ becomes strongly anisotropic, which does not seem to be the case of Bi_2_Se_3_^20, 21^. This formula is valid only for systems with well-defined effective masses, and therefore strictly parabolic bands. Nevertheless, it is the existence of the direct band gap (implied by our PL data), which ensures such a parabolicity of bands, at least in the vicinity of the band edges. Let us also note, the parabolic shape of both, conduction and valence, bands is also consistent with results of magneto-transport experiments performed on bulk Bi_2_Se_3_ specimens^20–23^ and Landau level spectroscopy on thin epitaxial layers^11^.

**Figure 3:**
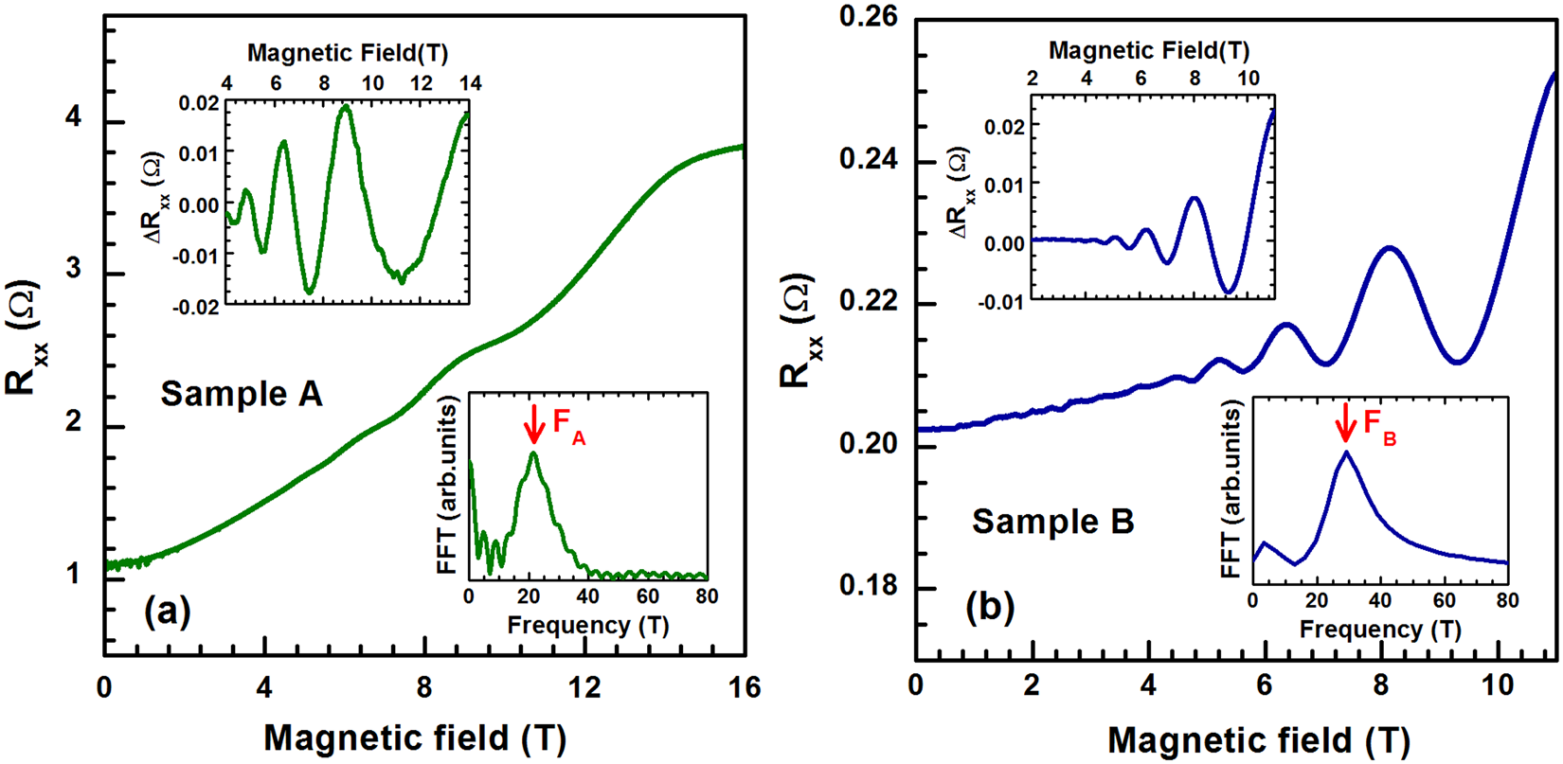
(**a**,**b**) Magneto-resistance data on samples A and B taken at the temperature of *T* = 4.2 and 1.4 K, respectively. The upper and lower insets show background-removed data Δ*R*_*xx*_ and fast Fourier transform of Δ*R*_*xx*_(*B* ^−1^), respectively. The latter imply the oscillation frequencies *F*_*A*_ = 22.0 ± 0.5 T and *F*_*B*_ = 28.0 ± 0.5 T for the samples A and B, respectively.

Importantly, the Moss-Burstein shift may be expressed as Δ*E*_MB_ = *eħF*/*μ*, where *μ* stands for the reduced mass, *m*_*e*_ *m*_*h*_/(*m*_*e*_ + *m*_*h*_), and *F* is the characteristic frequency of the 1/*B*-periodic quantum oscillations, *F* = *m*_*e*_ *E*_*F*_/(*ħe*), which are associated with the Landau quantization of electrons, emerging under an externally applied magnetic field. These were clearly resolved in the magneto-transport data in a form of Shubnikov-de Haas effect (Fig. 3a and b). In both samples, a single oscillation frequency has been found, *F*_*A*_ = 22.0 ± 0.5 T and *F*_*B*_ = 28.0 ± 0.5 T, consistently with expectations for a degenerate electron gas in a simple parabolic conduction band. Let us note that the observed Shubnikov-de Haas oscillations originate in bulk states of Bi_2_Se_3_. The contribution of the surface states to the transport response remains at given bulk electron densities negligible.

This may clearly demonstrated, *e*.*g*., by the angle dependence of Shubnikov-de Haas oscillations, which we have studied on samples coming from the same batch in the scope of our preceding NMR study, see ref. 23 and the related Supplementary Materials. These magneto-transport experiments also implied (via damping of Shubnikov-de Haas oscillations with temperature) the effective mass of bulk conduction band electrons, *m*_*e*_ ≈ 0.12*m*_0_, which agrees well with values from previous studies^20, 22, 24–26^. Combining this electron mass with the hole mass from our recent magneto-transport studies performed on *p*-type Bi_2_Se_3_^21^, *m*_*h*_ ≈ 0.24*m*_0_, we obtain the reduced mass of *μ* ≈ 0.08*m*_0_, in perfect agreement with the value read directly from the separation of interband inter-Landau level resonances observed in our recent magneto-optical study^11^.

Taking account of the Shubnikov-de Haas oscillation frequencies and the estimated effective reduce mass, we get the Moss-Burstein shifts of ${\rm{\Delta }}{E}_{{\rm{MB}}}^{A}\approx 30$ meV and ${\rm{\Delta }}{E}_{{\rm{MB}}}^{B}\approx 40$ meV for the sample A and B, respectively. Subtracting these values from the optical band gaps ${E}_{g}^{\text{opt},{\rm{A}}}$ and ${E}_{g}^{\text{opt},{\rm{B}}}$, we obtain at the energy band gap of Bi_2_Se_3_: *E*_*g*_ = (220 ± 5) meV. This value is in perfect agreement with the PL results, when the simplest scenario of direct band-to-band recombination of free electrons and holes is considered. Let us also note that we do not consider the band gap renormalization, which for electron densities close to 10^18^ cm^−3^ provides us with a correction of the band gap well below the estimated error bar^27^.

Let us emphasize that the extracted band gap, *E*_*g*_ = (220 ± 5) meV, as well as its direct nature clearly contrasts with conclusions of several ARPES experiments performed on bulk Bi_2_Se_3_^3–5^, nevertheless not with all of them, see, *e*.*g*., refs 6 and 7 This technique thus seems to be well-suited for investigations of surface properties and the observation of conical bands on the surface of topological insulators is definitely one of its greatest achievements^28^. At the same time, the visualization of a truly bulk electronic band structure, especially in narrow gap materials, which are characterized by pronounced band bending effects^29^ and charge accumulation layers on the surface, may be a challenging task for the surface-sensitive techniques, with a strongly limited penetration depth.

## Conclusions

In summary, we have explored bulk Bi_2_Se_3_ using magneto-transport and infrared spectroscopy techniques aiming at determining the nature and size of the energy band gap in this 3D topological insulator. We have shown that the energy band gap is direct and falls into the interval of *E*_*g*_ = (220 ± 5) meV.
